# Treatment Trends in Dry Eye Disease and Factors Associated with Ophthalmic Follow-up Discontinuation in Japan

**DOI:** 10.3390/jcm8081120

**Published:** 2019-07-28

**Authors:** Miki Uchino, Norihiko Yokoi, Motoko Kawashima, Yamanishi Ryutaro, Yuichi Uchino, Kazuo Tsubota

**Affiliations:** 1 Department of Ophthalmology, Keio University School of Medicine, Tokyo 160-8582 Japan; 2 Department of Ophthalmology, Kyoto, Prefectural University of Medicine, Kyoto 606-8566, Japan

**Keywords:** dry eye disease, ophthalmic follow-up discontinuation, epidemiology, web-based survey

## Abstract

Despite the importance of dry eye disease (DED) treatment, the rate of DED treatment discontinuation, especially discontinuation of ophthalmic follow-up, remains unknown. This study aimed to assess the prevalence and risk factors of ophthalmic follow-up discontinuation for DED. A cross-sectional survey of 1030 participants was conducted using a self-administered web-survey instrument. We collected lifestyle information, history of DED diagnosis, types of treatment, frequency of eye-drop usage, symptoms, and the reasons for discontinuing treatment. Statistical analyses including logistic regression were used to evaluate the risk factors of discontinuing ophthalmic follow-up for DED. A past history of clinical DED diagnosis was reported by 155 (15.0%) subjects. Of those, 130 had persistent DED, and 88 (67.7%) of the subjects reported discontinuation of ophthalmic follow-up for DED. The most prevalent reasons for ophthalmic follow-up discontinuation were time restrictions, followed by dissatisfaction with the DED treatment. Duration after DED diagnosis was the only significant risk factor for discontinuing ophthalmic follow-up after adjusting for age and sex (odds ratio = 1.09, 95% confidence interval = 1.02–1.17, *p =* 0.009). In conclusion, longer DED duration after diagnosis was a significant risk factor for discontinuing ophthalmic follow-up for DED. This study showed that DED ophthalmic follow-up discontinuation involves both medical and non-medical reasons. Clinicians need to be aware of them, and preventative effort is needed to avoid discontinuation.

## 1. Introduction

The prevalence of dry eye disease (DED) continues to rise due to several psychosocioeconomic factors, including an increasingly digitalized world, an aging society, and stressful social environments [1,2]. DED may cause ocular surface damage, eye discomfort, and impaired vision, but it can also lead to substantial economic problems due to decreased quality of life and work productivity [3,4]. The economic burden to the health-care system is particularly related to indirect costs, such as those attributed to reductions in working hours and in effectiveness due to dry eye symptoms [4]. The worldwide prevalence of DED ranges from approximately 5% to 50% and has been shown to be dramatically higher in people aged 50 years or older [5]. As the population ages, establishing effective treatment strategies and ensuring adherence to the treatment for DED become increasingly important.

The treatment options for DED have been rapidly increasing, and treating clinicians as well as patients with DED can select from a variety of methods, including tear drops, gels, ointments, and punctal occlusion [6]. However, there are marked reported discrepancies in the degree of DED severity and treatment response between patient and clinician assessments, which have been found to result in discontinuation of ophthalmic follow-up among patients with DED [7].

Treatment discontinuation has been recently studied. In the fields of diabetes mellitus (DM) and psychiatry, the rate of treatment discontinuation has been reported to be relatively high, at 11.1% to 53% [8,9]. There is increased interest in studying the effects of medication adherence on health outcomes. However, if patients appropriately stop treatment because of side effects and treatment failure, it is neither possible nor clinically meaningful to estimate the effect of full medication adherence.

Since the discontinuation of treatment might result in negative outcomes, the Ministry of Health, Labor and Welfare has established a manual for preventing treatment discontinuation in DM [10]. However, the prevalence and reasons for discontinuation of ophthalmic follow-up among patients with DED remains unknown. Therefore, the purpose of this study was to examine the frequency of DED ophthalmic follow-up discontinuation and to identify specific risk factors or characteristics related to discontinuation.

## 2. Materials and Methods 

### 2.1. Study Population

We distributed a web-based survey with a self-screening questionnaire on dry eye symptoms. Subjects who were 20 years of age or older and enrolled to the web survey panel (Macromil Incorporated, Tokyo, Japan) were asked to participate in this study. Among 1,200,000 panels, 5000 participants who used a visual display terminal (VDT) during work were randomly selected. We distributed an invitation mail to 5000 participants without introducing the aim of the study, and the first 1030 to respond were enrolled in this study. The study took place from 24 April to 25 April 2018. Each participant received USD 60 cents as compensation. 

Regarding the DED status, we included questions on the history of DED diagnosis, duration after DED diagnosis, current DED treatment including the name of the product, frequency of product use, current checkup status with the treating physician, current DED status (cured or persistent), if the patients had discontinued the treatment, and the specific reasons for DED ophthalmic follow-up discontinuation; we also included the Dry Eye-related Quality of life Score (DEQS) [11]. The status of DED was defined as “cured” or “persistent” depending on the clinician’s diagnosis, and “discontinuation of treatment” was defined as the patient having voluntarily stopped visiting the physician.

The following data were also collected: age, sex, household income, residence, marriage and child status (yes and no), smoking status (current smoker or not), and use of contact lenses (CLs). 

Based on our previous studies [1,2], we defined the duration of VDT use (stratified, none to longer than 10 hours in 1-hour categories). 

### 2.2. Ethical Statement

The study was conducted in accordance with the ethical principles of the Declaration of Helsinki and was based on a protocol approved by the Institutional Review Board of the Haneginomori Eye Clinic. Informed consent, including approval for the use of information collected during the study, was obtained from the participants through the survey website.

### 2.3. Dry Eye Symptom Questionnaire

The symptoms of each subject were assessed using the DEQS questionnaire. The DEQS questionnaire was recently developed in Japan, and its internal consistency, test–retest reliability, discriminant validity, and responsiveness to change have all been validated. The DEQS consists of 15 questions that assess the impact of DED on daily life and any irritating ocular symptoms. The summary scale score ranges from 0 (best) to 100 (worst) [11]. 

### 2.4. Specific Reasons for Discontinuing DED Treatment

The reasons for discontinuing DED treatment were collected in detail using the following question: “What was the reason for DED treatment discontinuation, especially discontinuing ophthalmic follow-up?” Answer options were provided in multiple-choice format, and the respondents were allowed to select all applicable options from: (1) “too busy to visit the physician,” (2) “long waiting time at the clinic,” (3) “symptom was not relieved by treatment,” (4) “high out-of-pocket cost,” (5) “unsatisfied with the treatment,” and (6) “insufficient explanation from the physician.”

### 2.5. Details of the DED Treatment

Details regarding the acquisition of eye drops were evaluated using a single question: “Where do you obtain your eye drops?” The possible answer options were: (1) drug store over the counter, (2) prescribed by a physician, (3) no eye drops obtained. Finally, we inquired regarding the type of eye drops using one question: “What is the type of your eye drops?” The possible answers were: (1) artificial tears (saline only), (2) hyaluronic acid, (3) diquafosol sodium, (4) rebamipide, (5) steroid, and (6) unknown. The answer options were provided in multiple-choice format, and the respondents were allowed to select all applicable.

### 2.6. Statistical Analysis

Fisher’s exact test, the Mann–Whitney U-test, and *t*-test were used to compare parameters between subjects in the DED and non-DED groups. Using a logistic regression model, we calculated odds ratios (ORs) and 95% confidence intervals (CIs) of the discontinuation of ophthalmic follow-up for DED in association with demographic, lifestyle, and medical factors. First, we carried out univariate analyses of the associations between each factor and discontinuation of ophthalmic follow-up for DED. Then, mutual adjustment for all associated factors identified in these univariate analyses (*p* < 0.2) was performed. In order to examine the risk of discontinuation of ophthalmic follow-up for DED, we excluded patients whose DED had been cured. All statistical analyses were performed using STATA software, version 13.0 (Stata Corp, College Station, TX, USA). A *p*-value of <0.05 was considered to be statistically significant.

## 3. Results

### Characteristic of the Study Participants

All 1030 subjects who consented to participate completed the study, including 729 men (70.8%) and 301 women (29.2%). The mean (±standard deviation) age was 49.6 ± 0.4 years for men and 40.2 ± 0.6 years for women. 

Regarding the DED prevalence, 155 subjects reported having a history of diagnosed DED. Among them, 24 subjects (15.5%) reported a cured DED status, whereas 84.5% reported persistent DED. More than two-thirds of the patients with persistent DED reported having discontinued their DED treatment (Table 1).

In the comparison between patients who had continued and those who had discontinued treatment, those who had discontinued were significantly younger (continued vs. discontinued: 48.3 ± 1.5 years vs. 44.6 ± 1.0 years, *p =* 0.04, Table 1), more likely to be women, and had longer duration of having DED (*p =* 0.009). Although eye-drop use was more frequent and DED symptoms were more severe in patients who discontinued treatment, the difference between the groups was not statistically significant. For the acquisition of the eye drops, there was a significant difference between the treatment continuation group and the ophthalmic follow-up discontinuation group (both *p*-values < 0.01). The current eye drop use showed a significant difference among the three groups (Table 1). Forty percent of the treatment continuation group was using eye drops prescribed by a physician; however, only 10% of the ophthalmic follow-up discontinuation group was using prescribed medicine. The most common method of eye-drop acquisition was over the counter.

In the persistent DED group, the most common eye-drop type used was hyaluronic acid, along with artificial tears. Half of the participants in the DED cured group were using artificial tears that were obtained over the counter. 

The specific reasons for discontinuing ophthalmic follow-up were evaluated (Table 2). More than two-thirds of patients complained that they lacked the time to visit the physician (71.6%) and of long waiting time in the clinic (35.2%). For the treatment satisfaction category, 35.2% of the patients stated that their symptoms persisted despite the treatment, 20.5% reported dissatisfaction with the treatment, and 8% complained that the physician’s explanation of the treatment was insufficient. Finally, 25.0% stated that the cost of the treatment was too high (Table 2).

Next, the risk factors for ophthalmic follow-up discontinuation were evaluated. The univariate analysis showed that sex and duration of DED treatment were associated with discontinuing ophthalmic follow-up for DED (Table 3). After controlling for age and sex, we found that each additional year of DED treatment increased the risk by 9% (OR = 1.09, 95% CI, 1.02–1.17).

## 4. Discussion

In this study we found that the prevalence of discontinuing ophthalmic follow-up among patients with DED is high in the Japanese population. To the best of our knowledge, this was the first study to evaluate the prevalence of discontinuing ophthalmic follow-up among patients with DED in Japan. Our study revealed that the prevalence of discontinuing ophthalmic follow-up was 67.2%, which was considerably high compared with other diseases [8,9]. The rate of treatment discontinuation for DM has been reported at 11.1% and at 53% for psychiatric disorders [8,9].

As many studies have reported, DED is a chronic disease whose worldwide prevalence reportedly varies from 5% to 50% and is particularly prevalent among women, older individuals, and the Asian population [5]. This high prevalence might play an important role in not considering DED as an important disease for physicians to prioritize its treatment. In addition, it might lead patients to underestimate the importance of treating the disease.

In the field of DM, it has been noted that some patients ignore the importance of treating the disease. In the report of the Ministry of Health, Labor and Welfare, it was stated that those at higher risk of DM treatment discontinuation were full-time male workers, younger than 50 years, especially those in their 20s, and the patients who had a history of discontinuation, regardless of the severity of DM control. The time required for the treatment, its cost, and underestimation of its importance have been cited as reasons for treatment discontinuation [10]. 

In our study, we also found specific reasons for ophthalmic follow-up discontinuation. To our surprise, the reasons were not only personal, but also treatment related. Regarding personal reasons, more than 70% of patients reported lacking the time to commit to the treatment process. Specifically, there were two primary time-related concerns; one was that patients reported a lack of time to visit the physician, and the other was the long waiting time at the clinic. The first reason was twice as frequently reported as the second, which suggests that patients do not prioritize their DED treatment over their other obligations.

Interestingly, more than one-third of the subjects stated that discontinuing ophthalmic follow-up resulted in a lack of improvement of their DED symptoms contrary to expectations of their treatment. In addition, 20.5% complained of dissatisfaction with their treatment and insufficient explanations from the physicians. As there are numerous types of ophthalmic eye drops and methods to treat DED, different physicians select different methods. The correlation between dry eye tests and symptoms of discomfort is generally weak because of the wide-ranging etiologies of DED and the great variability of clinical signs [12]. In 2017, a standard clinical test was established for DED diagnosis in the DEWS II report; however, not all diagnostic tests can be performed worldwide [13].

There is a recommended treatment method in the DEWS II report [14]; however, the availability of certain drugs differs among countries, and thus there is no global standard to follow for DED treatment. In addition, some treatments such as autologous serum eye drops are not available in many hospitals, which precludes providing uniform treatment to patients. 

Yeh et al. reported that there is a discrepancy between patient and clinician assessments of DED treatment [7]. With a sample of 466 subjects, the agreement between clinician and patient assessments in terms of disease severity and treatment response was significantly low. They reported that clinicians might underestimate DED severity and the persistence of dry eye symptoms after treatment with artificial tears [7].

This discrepancy between physicians and patients regarding DED severity might result in insufficient explanation from the physician. Since the physicians estimate the patients’ eye condition as not very severe, they might not provide a detailed explanation. In our study, we found that 8.0% of patients considered that the explanation they received from the clinician was insufficient. This could also lead to discontinuing ophthalmic follow-up. 

Our study also revealed that a 1-year increase in the duration after diagnosis of DED increased the risk of discontinuing ophthalmic follow-up by 9%. To the best of our knowledge, no study has evaluated the risk of duration after diagnosis and the rate for ophthalmic follow-up discontinuation.

As is the case for DM treatment, we found that one-quarter of the patients reported that high out-of-pocket cost was one of the reasons for DED treatment discontinuation. Yamada et al. reported that, in Japan, the annual average DED treatment cost is estimated at 323 ± 219 US dollars for the pharmacological cost and at 165 ± 101 US dollars for the clinical examination cost. The total direct costs including those for punctual plug occlusion amounted to 530 ± 384 US dollars [15]. Conversely, the Japanese national tax agency reported that the Japanese average annual income in 2017 was 52,000 US dollars for men and 27,000 US dollars for women [16]. Overall, compared with the annual income, the treatment cost of DED is relatively high. We considered that this high expense may be one of the reasons leading to discontinuation of ophthalmic follow-up. 

This study also revealed that although many patients had selected to discontinue follow-up examinations, 10.2% were using prescribed medicine. Furthermore, 68.2% of patients were using eye drops that had been obtained over the counter. This tendency of using eye drops without visiting an ophthalmologist for a follow-up examination might be explained by the high cost of the clinical examination despite the long waiting time [15]. 

The strengths of this study include that it revealed specific reasons for discontinuing ophthalmic follow-up for DED. We found that time-related issues play an important role in ophthalmic follow-up discontinuation. Second, dissatisfaction with treatment also plays an important role in its discontinuation. The results of this study may provide guidance for specific precautions such as minimizing waiting time at the clinic and increasing the patient satisfaction rate. Ever since DED treatment started, numerous types of eye drops have been developed, which have enabled us to personalize treatment for each patient. However, the physician needs to understand that there is a discrepancy between the patient’s and physician’s understanding of this disease; therefore, an effort to bridge this gap is important.

We acknowledge several limitations to this study. First, there were significant sex differences. In order to evaluate the real prevalence of ophthalmic follow-up discontinuation, it might be meaningful to include subjects matched for sex and age to avoid selection bias. Second, DED diagnosis relied on self-reported questionnaires, and no clinical examination was performed at recruitment. Although we used a questionnaire that has been validated to evaluate the prevalence, no objective diagnostic testing of DED was performed. Another limitation of using a self-administered questionnaire, which might have introduced biases, pertained to possible misunderstanding regarding the answers related to medication use and the subjects’ health status. In addition, we did not obtain information regarding the status of menopause and use of hormone replacement therapy, as well as details on the medications used for systemic diseases such as hypertension. Furthermore, our compensation for this questionnaire was 60 cents, therefore it might have led to a bias in the participant population. Finally, this study was conducted among the general population, not among patients with DED; therefore, the participants with clinically diagnosed DED were relatively few. It is important to note that the study findings may not be generalizable. Currently, a second study with a relatively larger sample of participants with DED matched for age and sex is ongoing.

Although further studies are needed to increase our understanding of the significance of this important public health concern, the results of this study may increase awareness for DED evaluation. 

In conclusion, discontinuing ophthalmic follow-up for DED is prevalent in the Japanese population. Effort is required to shorten the waiting time at the clinic and improve the treatment satisfaction rate in order to reduce the rate of ophthalmic follow-up for DED. Importantly, we found that the longer the duration after diagnosis of DED, the greater the risk for discontinuing with follow-up examinations.

## Figures and Tables

**Table 1:** Characteristics of subjects according to the dry eye condition.

Variables	Dry Eye Remain (N = 131)		Dry Eye Cured	*p*-value	
	Treatment Continued	Ophthalmic Follow-up Discontinued		(Continue vs. Discontinue)	(Discontinue vs. Cured)
	(*n* = 43)	(*n* = 88)	(*n* = 24)		
Age (mean ± SD)	48.2 ± 10.3	45.3 ± 10.4	43.1 ± 11.8	0.04	0.38
Gender (Female)	16 (33.3%)	47 (53.4%)	12 (50.0%)	0.03	0.77
Marriage (Yes)	29 (60.4%)	50 (56.8%)	14 (58.3%)	0.68	0.89
Child (Yes)	25 (52.1%)	36 (40.9%)	9 (37.5%)	0.21	0.77
House hold annual income				0.61	0.31
Under $40,000	9 (18.8%)	17 (19.3%)	2 (8.3%)		
$40,001–$60,000	7 (14.6%)	19 (21.5%)	5 (20.8%)		
$60,001–$80,000	6 (12.5%)	14 (15.9%)	2 (8.3%)		
Over $80,001	26 (54.2%)	38 (31.8%)	15 (62.5%)		
Duration of having DED				0.10	0.72
under 1 year	13 (29.6%)	12 (13.6%)	7 (29.1%)		
2–4 years	10 (22.7%)	15 (17.0%)	6 (25.0%)		
5–8 years	8 (18.2%)	21 (23.9%)	3 (12.5%)		
Over 8 years	13 (29.6%)	38 (43.2%)	8 (33.3%)		
Year after diagnosis	5.8 ± 5.3	8.7 ± 6.8	5.5 ± 5.2	0.01	0.04
Frequency of eye drop	3.8 ± 1.7	4.1 ± 1.9	2.9 ± 1.0	0.31	0.01
Method of eye-drop acquisition				0.01	0.01
Over the counter	21 (48.8%)	60 (68.2%)	12 (50%)		
Prescribed by physician	18 (41.8%)	9 (10.2%)	0 (0%)		
No medication use	4 (9.3%)	17 (19.3%)	12 (50%)		
Type of eye drops				0.003	0.001
Artificial tears	9 (23.1%)	33 (47.8%)	6 (50%)		
Hyaluronic acid	16 (41.0%)	45 (72.5%)	0 (0%)		
Diquafosol sodium	7 (17.9%)	5 (7.2%)	0 (0%)		
Rebamipide	8 (20.5%)	4 (5.8%)	0 (0%)		
Steroids	4 (4.4%)	3 (1.4%)	0 (0%)		
Unknown	6 (6.6%)	5 (7.2%)	6 (50%)		
VDT hours (mean ± SD)	2.5 ± 1.1	2.6 ± 1.5	2.9 ± 2.1	0.29	0.18
DEQS	20.3 ± 14.0	20.9 ±13.2	11.8 ± 8.4	0.95	0.001

SD: standard deviation; DED: dry eye disease; VDT: visual display terminal; DEQS: Dry Eye-related Quality of life Score.

**Table 2:** The reasons for discontinuing ophthalmic follow-up.

Reason*	Number	(%*)
	(*n* = 88)	
**Personal reason**		
Too busy to visit physician	63	(71.6%)
**Treatment-related reasons**		
Long waiting time at clinic	31	(35.2%)
Symptoms do not change with treatment	31	(35.2%)
Out-of-pocket cost is too high	22	(25.0%)
Unsatisfied with the treatment	18	(20.5%)
Insufficient explanation from the physician	7	(8.0%)

* Multiple choice—the total will be over 100%.

**Table 3:** The risk factors for ophthalmic follow-up discontinuation.

Variables	Univariable	95% CI	*p*-Value	Multivariable	95% CI	*p*-Value
Age	0.97	(0.94–1.01)	0.096	0.97	(0.94–1.01)	0.189
Sex	2.29	(1.10–4.77)	0.023	1.84	(0.83–4.09)	0.136
Household income	0.87	(0.65–1.18)	0.368			
Years after diagnosis	1.09	(1.02–1.17)	0.007	1.09	(1.02–1.17)	0.009

CI: confidence interval.
